# Nitrous Oxide

**Published:** 1894-07

**Authors:** J. Lathrop


### Nitrous Oxide.

BY J. LATHROP, JR., D.D.S.

Read before the Thirty-eighth Annual Meeting of the Michigan Dental Association.

Nitrous oxide, or nitrogen monoxide, or better known as laughing gas, is a colorless, odorless gas at ordinary temperature; but is a clear liquid under pressure of fifty atmospheres at forty-five degrees F.

It was first discovered by Priestly in 1776, but its exhilarating and analgesic effects were not noticed until 1800, when Dr. Davy made them known. As an anæsthetic it was not used until 1845 when Dr. Horace Wells, a dentist, demonstrated its power of destroying physical pain, and from that time until the present it has been extensively used by our profession. It is without an equal in minor operations about the mouth, although it has been used in major ones; and patients have been kept under its influence an hour and forty minutes.

The gas can be obtained by reducing nitric acid and is oftentimes formed when copper is treated with the concentrated acid, but when it is made in this way we find so many impurities in it that it is hardly practicable. The usual method of making it, is by heating in a retort pure ammonium nitrate until it decomposes and the gas is liberated, but the heat should not exceed 400 F. for above that, bad gas is formed. The ammonium nitrate melts at 320 F. and the gas is given off, and very rapidly at 470 F. The ammonium nitrate breaks down into nitrous oxide and water.

We find many impurities passing over with the gas unless the ammonium nitrate is perfectly pure, chlorine being the principal one, and before the gas is let into the gasometer it should be passed through three receivers, the first of which contains water to cool the gas, the second potassium hydrate to neutralize any chlorine, nitric or nitrous acid or carbonic acid gas that may be present. The third contains ferrous sulphate to collect from the vapor any nitric oxide and reduce it to nitrous oxide.

We find that cool water will absorb about 80 per cent. of the gas but on warming it will give it up. The gas has a specific gravity of 1.527 and will support combustion almost as well as pure oxygen.

The gas thus obtained is one of our general anæsthetics, when inhaled in sufficient quantity will produce profound anæsthesia. It does this probably acting indirectly, by interfering with the circulation in the brain, and arresting the process of oxidation and tissue change in the nerve-cells, which is absolutely necessary for functi nal activity. The time for its anæsthetic effect to be produced varies, and no set time can be given. We have three distinct stages during anæsthesia of nitrous oxide: the excitant, narcotic and anæsthetic. We find it first affecting the heart and cerebrum by stimulation, producing the excitement or first stage. In the second stage, the cerebrum and reflexes are partially narcotized and the patient becomes quiet and indifferent, with pulse reduced to almost normal, breathing regular and deep, face somewhat flushed. The patient loses consciousness or inability to talk or think, but the reflexes and motor influences are active. In the third, or anæsthetic stage, the cerebrum and reflexes are abolished and the motor impulses impaired. The pulse is full and regular, breathing is periodic and interrupted ; more or less cyanosis appears in the face and other delicate and vascular parts of the integument, and there is no response to the irritation of the conjunctiva, which is one of the last reflexes affected. The gas is not decomposed in the body, but it is exhaled in the same form as inhaled. In the body it is found in the blood and takes the place of the oxygen. Nitrous oxide can not be decomposed in the system, since the temperature of the body is not high enough to destroy its molecular affinity, and there are no chemical affinities met with in the system sufficiently powerful to change it. Experiments also show that the exchange of gases practically stops after it has been administered for a few minutes, and the gas is exhaled in the same form and quantity as inhaled.

The blood of a dog was analyzed after the administration of the gas for one minute and forty-five seconds and was found to contain :

| | | | |
|---|---|---|---|
| $CO_2$ gas | 37. | 3 minutes ($CO_2$ | 36.6 |
| O | 5.2 | ( O | 3.3 |
| $N_2O$ | 28.6 | ($N_2O$ | 34.6 |
| N | .7 | | |
| 4 minutes ($CO_2$ | 34. | | |
| ( O | 5.05 | | |
| ($N_2O$ | 37. | | |

Like ether, it kills by paralyzing the center of respiration and not by asphyxia, for we do not have an over-amount of carbonic acid gas. The heart center is not paralyzed so quickly because of its characteristic stimulation even to the last.

From its first use until the present time there are only eleven deaths reported from the administration of nitrous oxide, and only five of these can be with any certainty traced to the direct action of the gas. In four only of these was a post mortem held, and three were caused by paralysis of the heart and lungs, while in the fourth the verdict was asphyxia.

It is the safest of all our anæsthetics, and in comparison with chloroform we find one death to every 2,500; and ether one death to every 23,000. It can be given to people of all ages and conditions with comparative safety except in fatty degeneration or valvular trouble of the heart, aneurism or atheromatous blood vessels, and to women after the seventh month of pregnancy.

We may have after or secondary effects from the use of nitrous oxide which do not demonstrate themselves for days after the operation, and it is probable many fatal cases will be traced to the after-effects of nitrous oxide, as there is already some evidence to confirm this prediction.

Dr. La Fount warns against the production of diabetes. He reports a case where sugar appeared in the urine and also in his own after the administration of the gas. Dr. Kendelheim died from diabetes which he persistently attributed to nitrous oxide.

While the after dangers of the gas are probably not great the high blood pressure with venous stasis, which are undoubtedly present during anæsthesia, strongly indicate that when atheroma or other diseases of the arterial walls exist nitrous oxide is less safe an anæsthetic than ether.

### ADMINISTRATION.

There are two forms of apparatus used for storing and administering the gas: one by the gas bag, the other the gasometer. The gasometer is almost universally used, it being the handiest can be used to a better advantage, for the gas can be better controlled than with the gas bag. It is also more economical and less liable to contamination. The only advantage the gas bag has over the gasometer is that it is portable.

In the administration of the gas to a patient, the mouth-piece and hood are used. Some practitioners use the mouth-piece, but the hood although it has some advantages is, without doubt, preferable. The operator has better control of the patient than when using the mouth-piece, unless he has a skilled assistant who will take charge of the administration. Many patients object to the mouth-piece because it seems unclean, from the fact that it has been in the mouths of many other people. The other features of the modern gas apparatus are so familiar as to need no further comment here. In brief, a good gas apparatus should be easily and quickly operated, easily kept in repair, of ample capacity and more or less ornamental, comely and tasteful, rather than formidable.

If the liquid gas is used the gasometer should always be kept nearly full, so that the gas may be of suitable temperature to administer at any time. When the gas is freshly released from the liquid state it is cold and not of suitable temperature to apply to sensitive mucous membranes.

Sufficient gas should be given to produce complete anæsthesia, for there is danger from shock and after-effects when complete ansæthesia is not produced. In complete anæsthesia the reflexes are paralyzed, and irritation of the sensory nerves by extraction of a tooth has no effect either upon the vagus or upon the vaso-motor ; while in imperfect anæsthesia the reflex centers of the heart and circulatory system may be paralyzed because of the presence of the gas in the blood, and the vagus centers not affected ; hence, the shock caused by the extraction of a tooth may, through a reflex action on the vagus or inhibitory center, cause the stoppage of the heart, there being nothing to counteract the tendency to faint, syncope may prove fatal. There is, however, less danger from the use of gas than with chloroform or ether, as the nitrous oxide causes a venous condition of the blood with consequent vaso-motor stimulation and contraction of the arterioles and a rise in blood pressure so that a tendency to syncope through vagus irritation is counteracted.

Strangulation is another thing that should be guarded against, for during the administration of the gas the tongue may fall back into the pharynx and the muscles of the pallet contracting thus causing a stoppage of the air passages.

During the administration the respiration should be regular, full and free; difficult, or irregular and spasmodic breathing are dangerous symptoms, so also is prolonged or indifferent breathing.

The pulse will be quick and nervous at first, but as the anæsthetic takes effect it becomes full, strong and frequent, finally becomes regular, full and a trifle more frequent than normal.

In giving the anæsthetic a third person should always be present, for we have a case on record where the operator was accused and convicted of a crime of which he was probably innocent.

Nitrous oxide is sometimes combined with ether or chloroform, or both, which prolongs anæsthesia, and when combined in this manner it is known as vitalized air.

On account of the brevity of the effect of nitrous oxide, more extensive operations are undertaken than are justifiable, resulting in severe nervous shock to the patient or bungling operations, causing more or less wasteful destruction of the oral tissues. It would be far better to repeat the administration, and attempt only so much work as may be properly done under each administration. There is usually no objection to a second and even a third administration at one sitting.

It is important that all valued convenient instruments and restoratives should be readily available. Proper and sufficient instruments for any possible emergency in difficult extractions are essential, even so are adequate restorative measures.

---

### DISCUSSION—CHLOROFORM, ETHER AND NITROUS OXIDE.

---

Dr. A. M. Long: I will say the paper just read is one of the best on nitrous oxide that I have seen. It is not an easy task to write on this subject; there is such a diversity of opinion and the experiments that have been made vary nearly as much as the different claimants vary as to its discovery. While there is a controversy as to the effects and methods and means of administering nitrous oxide, there shall be none between the author and myself in calling nitrous oxide gas an anæsthetic. There have been many circulars sent to dentists denouncing the anæsthetic effects and claiming that it is only "asphyxia." The author has pointed out the action of nitrous oxide on the nervous system and what nerves are mostly affected; also upon the heart and vascular system. He has also given the three different stages; excitement, narcotic and anæsthetic. In the excitement stage success depends largely on the operator and the surroundings. He must be composed and have perfect confidence in himself. Of course, we will take it for granted that he has a good gas apparatus and an inhaler, and that they are in perfect working order, and no air allowed to leak through the tube or inhaler, if air be allowed to mix with gas it will produce fear and often screaming. The effects and excitement of alcohol are quite different from that of air, it breaks out in a combative or pugilistic form. If I know that my patient has been drinking I refuse to administer gas until he is free from liquor. It takes but a step aside to increase or modify the action of gas. The inhaler valves must be large to admit the exhaling and inhaling, and they should be free from springs. The receiver should force the gas to the patient and it should be as easy to inhale as pure air. Inhalers with small inhaling and exhaling valves, and the patient compelled to draw the gas to him with lung force, will produce an unpleasant effect in the excitement stage, and, in fact, will, all through the administration, and a failure will be the result. The small valved inhalers that we find in our markets are more to blame for the suffocating effects, and the deep and irregular breathing, also the blueness which appears in the face and lips, than the gas.

We will admit, from the effects of the gas on the system, that there is some cyanosis visible, but I here repeat, that it is not so well defined where a perfect gas apparatus is in use.

On page 2, I see the author accepts the theory of the gas replacing the oxygen. I find but two principal theories in regard to the physiological action of nitrous oxide. One theory is that the anæsthetic effects are due to the greater volume of oxygen in the lungs than they are accustomed to receive. The nitrous oxide contains more oxygen in action, which produces a stimulating effect.

The other theory is, of insufficient oxidation; claiming that the gas acts principally by occupying the space in the lungs normally held by the oxygen of the air, and to its exclusion, preventing the proper oxidation of the blood followed by suspended sensation.

On page 6, the author refers to the combining of chloroform and ether with gas. I have, for over ten years, used equal parts of pure alcohol and chloroform—about two drops of the mixture to one gallon of gas.

I know there is a strong prejudice with many against the mixture, but I know where the combination has been tried it has grown in favor. It has been argued that each anæsthetic agent possesses properties peculiar to itself. We claim for it, that the powerful agents are so small in combination with the quantity of gas that the gas is not impaired by it, and that the chloroform does not get further than the first stage of stimulation before the gas has produced full anæsthesia. I prefer chloroform to ether, as it conforms more easily to nitrous oxide, and is less irritating.

G. E. Corbin, St. Johns: In relation to the profession of medicine and surgery and the profession of dentistry a question of more importance than the one now before us could hardly be raised. Just think for a moment what surgery was before the days of chloroform and ether. Think of calling in the neighbors when some one had been so unfortunate as to need a limb amputated—calling in several of the strongest men to lash the unfortunate person to the table, while amid the shrieks and groans of the sufferer the limb was amputated. Then think of the possibilities of the present day—of accomplishing all that while the patient is quietly sleeping, and perhaps enjoying pleasant dreams. But while this possibility of performing operations without pain has worked great benefit in some departments of the profession, it is also true that in others it has worked great injury, and I am sorry to say this latter fact is especially true of our own branch of the profession. In serious surgical operations we can well afford to take the risk of the legitimate effects of an anæsthetic; in trifling operations I think we cannot afford to take such risk. Hundreds and thousands of teeth that might have done good service, teeth that were perhaps better than those with which they were replaced, have been extracted simply because it could be done without pain.

During my early student days, at the University of Michigan from 1852 to 1855, I saw chloroform used freely by Dr. Gunn, Professor of Surgery at that time; I do not know that very much was known about ether then, at any rate chloroform was the anæsthetic generally used. At Blakely College during the winter of 1868 I saw many operations performed by the distinguished Dr. Gross, and the anæsthetic used was almost always chloroform. It was administered by saturating a towel with chloroform and then pressing the towel tightly over the mouth and nostrils of the patient. Sometimes the patient would exhibit alarming symptoms; he would be rolled over and restoratives applied; the towel would then be placed over his mouth as before. Often it would be necessary to partially revive the patient two or three times before it was considered safe to completely anæsthetize him, but I saw no serious results arising from the use of this anæsthetic. The percentage of deaths as given in the first paper read this evening is certainly very serious and we regret it very much. Yet, beyond all question, in many of those cases had the operation been performed without an anæsthetic there would have been many more deaths caused by the shock of the operation. I am of the opinion that if chloroform be administered carefully, that is, diluted sufficiently and given slowly so as not to give any more than is needed to produce insensibility, there is as little danger as from the use of ether. If the statement in the first paper, that the anæsthetic effects of ether last longer than those of chloroform be true, it would seem to me that chloroform is the less dangerous, because the vital organs are not acted upon for so long a time. The danger is that one is very apt to give too much chloroform. Of course the effect of this anæsthetic upon the kidneys and other organs will alter the truth of my general remarks, but so far as its local effect, upon the respiratory organs is concerned, I think chloroform is much less harsh than ether.

Another point: Many times death is caused by strangulation. I remember Dr. Gunn used to refuse to administer an anæsthetic in cases where the operation would fill the mouth with blood. He considered there was more danger from strangulation than from the legitimate effects of the anæsthetic. Even in quite serious operations about the mouth he utterly refused to administer anæsthetics.

Dr. E. T. Loeffler, Saginaw: For the first year or two of my practice I used chloroform; it worked quickly and seemed perfectly satisfactory. But as I looked into the matter I changed my mind and I now am greatly in favor of using ether, especially when patients cannot be put in a horizontal position; I think dentists are always justified in using ether on account of the position of the patient.

We, as professional men, have a great responsibility in administering anæsthetics. Patients come into our offices in a specially sensitive condition. The parts to be operated upon are in an irritated condition and the whole nervous system is sympathetically affected, and that is a cause of great danger. We ought, on that account, to be very careful in administering an anæsthetic. Freedom of respiration should always be looked after. Too often patients are hurried into the chair without due precaution being taken. This point of precaution cannot be too strongly urged.

W. P. Morgan, Saginaw: I do not know that I can add anything to what has already been said. The ground seems to have been very thoroughly discussed. So far as the results of anæsthetics are concerned, I would rather take the chances of the effect of an anæsthetic than that of shock without it. One important point is to gain the confidence of your patient. If the patient thinks the operator is afraid, he, too, will be afraid, and this element of fear will make the danger much greater. Allow no one in the room to talk and disturb the patient. Before the anæsthetic is administered the patient should be shown how to breathe. Strangulation is usually caused because the breathing is not done in the right way. If short breaths are taken, after one or two it is necessary to take a long breath, and that is what causes strangulation; but if the breathing is done naturally, there need be no trouble. Just before administering the anæsthetic speak a few quieting words to keep the patient's attention away from what is about to be done. I consider it necessary to give the anæsthetic in a warm room, and the windows should not be opened lest the patient be in a draught. The sitting posture is safer, as there is less danger from strangulation.

Dr. Long: We all, I think, believe the introduction of anæsthetics to have been one of the most brilliant events in our history, and we all believe in their use. While nitrous oxide is considered one of the minor anæsthetics it is not one of the least; I think it one of the greatest and one of the safest to use.

Dr. D. Woolsey, Battle Creek: I would like to ask Dr. Corbin what effect chloroform would have upon children from six to ten years of age?

Dr. Corbin: I have administered chloroform to children of all ages and for other purposes than the extraction of teeth—for operations upon the ears and nose. It is only necessary to bear in mind what effect is desired and then to administer the anæsthetic so slowly that such effect can be attained. I cannot conceive why chloroform is not just as safe as any other anæsthetic that produces complete anæsthesia. I cannot but feel that there is some danger in every case of insensibility produced artificially. The danger is in saturating the system. Chloroform is so much more powerful than ether that, as I stated before, one is liable to give too much.

Dr. Woolsey: Children we cannot reason with, and when they are suffering we can do nothing with them, and my question was, whether or not the dentist would be justified in giving a child just a few whiffs of chloroform and in this way get over the difficulty?

Dr. Corbin: I think just enough chloroform to quiet the child, to produce unconsciousness, would be justifiable. Of course, the less of the anæsthetic given the better.

Dr. W. H. Jackson, Ann Arbor: I would like to ask if nitrous oxide is a chemical compound? Does it give up its constituent parts or does it go into the blood unchanged?

Dr. Lathrop: There is a difference of opinion as to that.

We used to think that part of the nitrous oxide was assimilated by the system. We, too, used to think that lime when taken went directly to the teeth. I believe that theory has changed and authorities now state that we do not get a direct effect from the lime, but that it must be taken through the stomach in a vegetable form. I think it is more generally believed now that the blood does not take up the gas; it is simply exhaled as pure air.

Dr. Jackson: In nine cases out of ten, where death occurs after the administration of anæsthetics, it arises from accident rather than from the effects of the anæsthetics. The attention of the assistant is often too much centered upon the operator, when it should be centered upon the patient. Many times in my own practice have I been obliged to stop and run my forceps into the throat and pull out the tongue to prevent a fatal accident, the cause of which would, in all probability, have been attributed to the anæsthetic. Then, too, we are apt not to pay enough attention to the clothing; it should be loose enough not to impair, in any way, the breathing. A careful examination of the condition of the patient should always be made, because a little overstraining of the nerves at the time of administering the anæsthetic may cause fatal results. There are three sets of nerves acted upon—the nerves of sensation, the nerves of voluntary motion and the nerves of involuntary motion. In the susceptibility of those nerves to the anæsthetic lies the danger. If the condition is such that the nerves of involuntary motion are acted upon first, where is your patient? Where are you? And there is a possibility of such a condition being met with. It is well to have a third person present when an anæsthetic is administered. Should time of danger come it needs a cool mind—a moment's hesitation may be fatal.

Dr. W. W. Williams, Sault Ste. Marie: I had, perhaps, a somewhat unusual experience with one case. I kept a patient alive for three weeks longer than he would otherwise have lived by the administration of nitrous oxide. The patient had a serious disease; he had considerable trouble with his heart and was expected to drop away at any moment. In the meantime he suffered with toothache. He came to me to have them extracted.

I administered gas and extracted two teeth. He went away from the office feeling better than he had for some time before; a condition which he attributed to the effects of the nitrous oxide. After that, whenever he felt worse than usual, he would come to me to have a little gas administered. He did this, at intervals, for three weeks; he then went away into the country; while there, after eating a hearty dinner, he was taken very sick and died before he could reach home. His friends thought if he only could have had some gas administered then he would not have died.

Dr. J. Lathrop, sr., Detroit: I am a believer in nitrous oxide. Of the two modes of administering it each has its advantages. That employing the mouth-piece keeps the mouth open when the patient is under the influence of the anæsthetic, though I, personally, have experienced little trouble in getting into the mouth of the patient after he has been anæsthetized. With the hood I have had a little trouble. Some dentists complain of patients, during the first stages of anæsthesia, becoming excited. I have found it well, when there are signs of distress and nervousness, to admit a little air. Many times I have overcome this difficulty by requesting the patient to breathe rapidly, say fifteen inhalations, before putting the hood to the mouth; the patient will pass under the influence of the anæsthetic much quicker and less gas will be needed.

As to the use of cocaine and other modern appliances for producing anæsthesia, I think in nearly all cases nitrous oxide might be applied more advantageously. I have sometimes given what the patient supposed was nitrous oxide and extracted teeth without pain, so the patient claimed, when, in fact, nothing but ordinary air had been inhaled. This method certainly has its advantages; what its scientific effect is I am unable to tell; the results are admirable.

Dr. H. P. Ball: I administered nitrous oxide to a patient. Three days afterward she was taken sick and has been sick ever since. Her family physician claims the gas was the cause of her illness. I would like to ask if this could have been the case.

Dr. Jackson: That depends on the nature of the sickness.

It might have resulted from the operation. The blood clot may have broken down and blood poisoning have been the cause. If this was the cause you should have been called upon to treat it. Every young dentist should study enough of medicine to treat such cases as are liable to come under their hands. If the patient does not call upon you in such cases, you cannot be held responsible for the results. Dentistry is only a branch of medicine. You expect an aurist to treat not only your ears, but your general system ; a dentist should treat his patients constitutionally as well as locally.

Dr. Ball : I do not think the clot was broken down in this case; so far as I could determine the symptoms seemed to be such as would be caused by the gas. Some of the doctors say the patient was coming down with some systemic disease which came out at that time. But her family physician says it was caused by the gas.

Dr. F. W. Temple, Grand Rapids: A lady went to a dentist to have a tooth extracted. She was advised to take gas and assured that no harm would result. She took it ; there was considerable trouble in restoring her to a normal condition, but she finally came to and went home. Just as she was about to ascend the steps at her home she fainted. She was sick for four or five weeks. A corps of doctors attended her; she was delirious for two weeks ; but finally she recovered. What was the cause is not known. It is a question whether her illness resulted from the effects of the gas or from some systemic trouble.

Dr. Cleland, Detroit: I have had a few serious experiences with nitrous oxide. The gas had been kept where some drugs were stored and it had taken them up. A new retort was strongly impregnated with it.

In one case where the gas had probably taken up the odor of the drug, I did not notice this in the gas itself, but detected it in the patient's breath. She coughed so hard I was unable to use it; the cough was similar to that induced by sulphuric acid.

A Gentleman : I, too, have had an experience similar to that related by Dr. Cleland. At one time the gas had a peculiar odor, like that of chloride of lime. It caused coughing and irritation. Of course we did not use it.

With regard to patients being taken sick after the administration of gas, I think many such cases could be traced to some other cause than the anæsthetic. Many would have been sick had they not taken an anæsthetic. I have had patients who were sick after having teeth extracted, although no anæsthetic was administered.

PROF. J. O. REED, Ann Arbor: I would like to ask whether it is customary in dental practice to prescribe an antiseptic mouth-wash after operations about the mouth. If that were done it would not seem that bad results would so often follow. After the extraction of teeth the patient often has a high temperature and is obliged to go to bed. Could that not be avoided if an antiseptic wash were used ?

DR. JACKSON: After administering an anæsthetic, especially chloroform, we are more apt to have a breaking down of the blood clot and consequent suppuration, than without an anæsthetic. But in either case I always use as a mouth-wash after extracting teeth a solution of boracic acid; should the blood clot then break down I use the following: 2 pints metallic iodide, $\frac{1}{2}$ drachm carbolic acid, 3 ounces glycerine. I prepare this at my office. Apply in full strength in every socket. It will not blister.

